# Autologous stem cell transplantation (ASCT) for acute myeloid leukemia in patients in first complete remission after one versus two induction courses: A study from the ALWP of the EBMT

**DOI:** 10.1002/cam4.5039

**Published:** 2022-07-26

**Authors:** Arnon Nagler, Jacques‐Emmanuel Galimard, Myriam Labopin, Didier Blaise, William Arcese, Silvia Maria Trisolini, Depei Wu, Arnaud Pigneux, Gwendolyn Van Gorkom, Marie‐Thérèse Rubio, Tobias Gedde‐Dahl, Anne Huynh, Francesco Lanza, Norbert‐Claude Gorin, Mohamad Mohty

**Affiliations:** ^1^ Division of Hematology Sheba Medical Center Tel Hashomer Israel; ^2^ EBMT Statistical Unit Paris France; ^3^ Department of Clinical Hematology and Cellular Therapy, Saint‐Antoine Hospital, AP‐HP Sorbonne University Paris France; ^4^ Sorbonne University, INSERM, Saint‐Antoine Research Centre Paris France; ^5^ Programme de Transplantation & Therapie Cellulaire; Centre de Recherche en Cancérologie de Marseille Institut Paoli Calmettes Marseille France; ^6^ Rome Transplant Network Università Tor Vergata Rome Italy; ^7^ Hematology, Department of Translational and Precision Medicine Sapienza University Rome Italy; ^8^ First Affiliated Hospital of Soochow University, Department of Hematology Suzhou China; ^9^ Service d'Hématologie et Thérapie Cellulaire, CHU Bordeaux Bordeaux France; ^10^ University Hospital Maastricht, Department of Internal Medicine Hematology/Oncology Maastricht The Netherlands; ^11^ Department of Hematology, Brabois Hospital Centre Hospitalier Régional Universitaire (CHRU) Nancy France; ^12^ Department of Hematology, Institute of Clinical Medicine University of Oslo and Oslo University Hospital‐Rikshospitalet Oslo Norway; ^13^ Hematology Department Institut Universitaire du Cancer Toulouse‐Oncopole Toulouse France; ^14^ Romagna Transplant Network Ravenna Italy

**Keywords:** acute myeloid leukemia, autologous, complete remission, induction chemotherapy, stem cell transplantation

## Abstract

**Background:**

Achieving complete remission (CR) is the main goal in AML treatment and a prerequisite for successful autologous stem cell transplantation (ACT).

**Methods:**

Comparing results of peripheral blood ACT in patients with AML in CR1 attained following 1 versus 2 chemotherapy courses transplanted in 2000–2019.

**Results:**

Patients 1532 (84%) with one and 293 (16%) patients with two induction chemotherapies courses (a total of 1825 patients) were included in the study. Follow‐up was 7.9 (95% CI: 7.4–8.4) and 7.7 (95% CI: 7.0–8.6) years (*p* = 0.8). Time from diagnosis to ACT was 4.7 (range, 3.9–5.8) versus 5.7 (range, 4.7–7.1) months (*p* < 0.001), respectively.

Leukemia free survival (LFS) and overall survival (OS) at 5 years were inferior for patients achieving CR1 with 2 versus 1 course of chemotherapy: 26.6% versus 41.7% (HR = 1.42 [95% CI: 1.22–1.66], *p* < 0.001) and 36.2% versus 53.3%, (HR = 1.48 [95% CI: 1.25–1.75], *p* < 0.001), and 5‐year relapse incidence (RI) was higher: 67.2% versus 52.3%, (HR = 1.46 [95% CI: 1.25–1.72], *p* < 0.001). Five‐year non‐relapse mortality (NRM) was 6.2% versus 6.0% for patients with 2 versus 1 chemotherapy courses, and did not differ significantly (HR = 1.31 [95% CI: 0.81–2.10], *p* = 0.27).

**Conclusions:**

LFS and OS were inferior and relapse rate was higher in AML patients who received two inductions chemotherapy courses to reach CR1 before being autografted. AML patients who required 2 induction courses to achieve remission, may be offered allogeneic transplantation rather than an autologous one in an attempt to reduce their high RI and improve outcomes.

## Graphical abstract

Autologous transplantation outcome in leukemic patients that achieve complete remission after two induction courses is inferior to outcome of patients achieving remission after one induction course. AML patients who need two induction chemotherapy courses to reach first complete remission may be offered allogeneic transplantation rather than autologous one aiming in reducing disease recurrence and improving transplantation outcome.

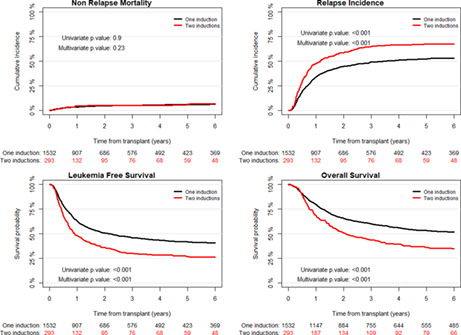

## 1 INTRODUCTION

Autologous stem cell transplantation (ACT) is a valid option for post‐remission consolidation for adult patients with favorable and possibly intermediate‐risk acute myeloid leukemia (AML).^1^, ^2^, ^3^, ^4^, ^5^ Besides the AML cytogenetics and molecular mutations, one of the prognostic factors with the highest significance for outcomes in patients undergoing any type of transplant and especially in those undergoing ACT is disease status at transplantation.^2^, ^4^, ^5^, ^6^, ^7^ The depth and quality of the remission assessed by both morphology and by the evaluation of measurable residual disease (MRD) are important prognostic factors in ACT for AML.^8^, ^9^, ^10^, ^11^, ^12^ In addition, the speed required to achieve remission and to clear the leukemic blasts may be important for the outcome and overall survival (OS) in patients with AML.^13^, ^14^, ^15^ The number of induction courses needed to achieve remission may be an additional surrogate marker both for leukemic risk as well as the quality of the remission in AML predicting response and transplantation outcome.^16^ In a previously reported study addressing patients undergoing allogeneic stem cell transplantation (HSCT), Lim SJ et al. analyzed the post‐transplantation outcome in 45 patients with high‐risk AML that achieved complete remission (CR) after 1–2 induction versus 3 or more induction courses pre‐HSCT from a matched sibling or unrelated donors and demonstrated a trend for better progression‐free survival and OS in the formers.^17^ An additional study was reported by Walter RB et al that analyzed 220 AML patients undergoing HSCT from human leukocyte antigen (HLA) matched donors demonstrating that patients who needed 2 chemotherapy courses to achieve a first CR (CR1) had an increased incidence of relapse translated into shorter relapse‐ free survival in comparison to their counterparts who needed only one chemotherapy course.^18^ Nagler et al have recently demonstrated in the Haploidentical setting that the relapse rate (RI) is significantly lower and the overall survival (OS) and leukemia‐free survival (LFS) significantly better in AML patients achieving CR with 1 versus 2 induction courses.^19^ It is conceivable that the number of chemotherapy courses required for CR1 achievement in AML is particularly important in the setting of AST since the graft versus leukemia (GVL) effect is not taking place in autologous transplantation and the success of the transplant procedure depends unconditionally on the depth of response and the chemotherapy mediated elimination of the leukemic stem/progenitor cells. Interestingly, in the historical era of ACT with marrow purged in vitro by mafosfamide, the efficacy of purging demonstrated by a reduction in relapse incidence post‐transplant was highly significant in slow and not rapid remitters.^15^

We thus performed a study to answer this question for the ACT and analyzed whether the results of ACT in AML patients transplanted while in the first remission would be better in those achieving CR1 after one induction in comparison to those achieving it only after a second chemotherapy course using the European transplant registry.

## 2 PATIENTS AND METHODS

### 2.1 Study design and data collection

This was a retrospective, multicenter study using the dataset of the acute leukemia working party (ALWP) of the EBMT. The EBMT is a voluntary working group of more than 600 transplant centers that are required to report all consecutive stem cell transplantations, outcomes and follow‐ups once a year. EBMT minimum essential data forms are submitted to the registry by transplant centers. EBMT centers commit to obtain informed consent according to the local regulations applicable at the time of transplantation in order to report data to the EBMT. Accuracy of data is assured by the individual transplant centers and by quality control measures such as regular internal and external audits. The results of disease assessments at transplant were also submitted and form the basis of this report.

Inclusion criteria were adult patients (≥18 years of age) at the time of transplant with AML in first hematological remission (defined as <5% blasts in the bone marrow)^20^ achieved following 1 or 2 inductions who underwent a first peripheral blood (PB) ACT between 2000 and 2019. Patients with secondary AML were excluded. Data collected included recipient characteristics (age, gender), Karnofsky performance score (KPS), disease characteristics, number of induction chemotherapy courses, disease status at transplant, year of transplant, details of the conditioning regimen, and doses of PB total nucleated cells (TNC) and CD34^+^ progenitors. For this study, all necessary data were collected according to the EBMT guidelines, using the EBMT minimum essential data forms. The list of institutions contributing data to this study is provided in the Appendix S1

Cytogenetic risk at diagnosis was categorized according to the cytogenetic abnormalities included in the 2017 European LeukemiaNet (ELN) recommendations for AML.^21^ Molecular abnormalities were not taken into account.

### 2.2 Statistical analysis

Median values and interquartile ranges (IQR) were used to describe quantitative variables, frequency, and percentage for categorical variables. The study endpoints were incidence of neutrophil recovery, non‐relapse mortality (NRM), RI, LFS, and OS. The primary outcome was the LFS. All endpoints were measured from the time of transplantation. Neutrophil recovery was defined as achieving an absolute neutrophil count of 0.5 × 10^9^/L for three consecutive days. OS was defined as time to death from any cause. LFS was defined as survival with no evidence of relapse. NRM was defined as death from any cause without evidence of relapse.^22^ The two patient cohorts (1 vs. 2 chemotherapy courses) were compared for the patient, disease, and transplant‐related characteristics using the Wilcoxon test for quantitative variables, and the chi‐squared or Fisher's exact test for categorical variables. The probabilities of OS and LFS were calculated using the Kaplan–Meier (KM) estimator. The incidence of neutrophil recovery, RI, and NRM were calculated using the cumulative incidence (CI) function in a competing risk setting, death without relapse being treated as a competing event for RI and relapse a competing event for NRM. Death and consecutive transplant were considered as competing event for the incidence of neutrophil recovery. Univariate analyses were performed using the log‐rank test for LFS and OS while Gray's test was used for CI. Multivariate analyses were performed using the Cox proportional‐hazards regression model. All variables differing significantly between the two comparison groups and known, or potential risk factors were included in the multivariate models. Results were expressed as the hazard ratio (HR) with a 95% confidence interval (95% CI). To test for a potential center effect, we introduced a random effect or “frailty” for each center into each multivariate model.^23^ Median follow‐up was calculated using the reverse KM method. All *p* values were two‐sided with a type 1 error rate fixed at 0.05. Statistical analyses were performed with R 3.4.1^24^ (available online at http://www.R‐project.org).

## 3 RESULTS

### 3.1 Patient, transplant, and disease characteristics

A total of 1825 patients met the inclusion criteria, 1532 (84%) received 1 and 293 (16%) 2 induction chemotherapy courses. The baseline demographic and clinical characteristics are demonstrated in Table 1. For patients receiving 1 versus 2 inductions pre‐ACT the median follow‐up from ACT was 7.9 (95% CI: 7.4–8.4) and 7.7 (95% CI: 7.0–8.6) years (*p* = 0.8), respectively. The median year of transplant was 2005 (IQR, 2002–2009) versus 2004 (IQR, 2002–2007), in the 1 versus 2 induction groups (*p* < 0.001). Median age was 49 (38–57) and 47 (36–56) years (*p* = 0.06) and 54% and 57%, respectively, were male (*p* = 0.35). The two patient cohorts differed significantly in their cytogenetic risk as patients with 1 induction were more frequently classified as a favorable risk in comparison to those with two inductions (18% vs. 14%) and had a lower percentage of adverse‐risk cytogenetics (6% vs. 13%), while 76% and 73%, respectively, had intermediate‐risk cytogenetics (*p* < 0.001). Cytogenetic risk classification was missing for 202 (11%) patients. A Karnofsky performance score (KPS) ≥ 90 was significantly more frequent in patients that received 1 versus 2 inductions (71% vs. 58% of patients) (*p* < 0.001). Time from diagnosis to ACT was 4.7 (IQR,3.9–5.8) versus 5.7 (IQR, 4.7–7.1) months (*p* < 0.001) for patients receiving 1 versus 2 inductions pre‐ ACT (Table 1). All patients were in CR1 at the time of ACT. All patients received a mobilized PB stem cell graft. The median cell counts were 7.5 (IQR: 4.2–12.3) versus 7.7 10^8^/kg (IQR: 4.4–12.2) (*p* = 0.74) for total nucleated cells (TNC) and 4.3 (IQR: 2.9–6.4) versus 4.1 10^6^/kg (IQR: 2.6–6.1) (*p* = 0.11) for CD34^+^ cells in patients receiving 1 versus 2 induction courses, respectively. For 873 (48%) and 366 (20%) patients TNC and CD34^+^ cell doses, respectively, were missing. The most frequent conditioning regimen in the two patient cohorts was Busulfan (Bu)/Cyclophosphamide (CY) 50% versus 45% and Bu/Melphalan (Mel) 17% and 19%, respectively, for induction groups 1 and 2, respectively. Conditioning was total body irradiation (TBI) based in 10% of each group. The 2‐year and 5 ‐year cumulative incidence of allogeneic transplantation consecutive transplant post‐AST was 20.8% (95%CI: 18.9–22.8) and 26.0% (95%CI: 23.9–28.1), respectively. For patients receiving only 1 induction, it was 19.8% (95%CI: 17.7–21.9) and 25.3% (95%CI: 23.0–27.6) and for patients receiving 2 inductions, 26.4% (95%CI: 21.4–31.8) and 29.6% (95%CI 24.3–35.1), respectively.

**TABLE 1:** Patient and disease characteristics

Clinical parameter	*N* = 1825	One induction (*N* = 1532)	Two inductions (*N* = 293)	Test *p*‐value
Age, median [IQR]	49.0 [37.7–57.3]	49.2 [38.3–57.4]	46.9 [35.7–56.4]	0.06
Patient sex				
Female	837 (45.9)	710 (46.3)	127 (43.3)	0.35
Male	988 (54.1)	822 (53.7)	166 (56.7)	
Year of transplant	2005 [2002–2009]	2005 [2002–2009]	2004 [2002–2007]	<0.001
Cyto AML classification				
Favorable	276 (17)	240 (17.6)	36 (14.1)	<0.001
Intermediate	1231 (75.8)	1045 (76.4)	186 (72.7)	
Adverse	116 (7.1)	82 (6)	34 (13.3)	
Missing	202	165	37	
KPS				
<90	314 (30.9)	245 (28.8)	69 (41.8)	<0.001
> = 90	703 (69.1)	607 (71.2)	96 (58.2)	
Missing	808	680	128	
Diag to TX in months, median [IQR]	4.9 [4–6]	4.7 [3.9–5.8]	5.7 [4.7–7.1]	<0.001
Conditionning regimen				
BuCy based	872 (49.2)	745 (50)	127 (45.4)	Not done
BuMel based	300 (16.9)	248 (16.6)	52 (18.6)	
Bu + Other	147 (8.3)	128 (8.6)	19 (6.8)	
Mel based	173 (9.8)	138 (9.3)	35 (12.5)	
TBI based	173 (9.8)	144 (9.7)	29 (10.4)	
Other	106 (6)	88 (5.9)	18 (6.4)	
Missing	54	41	13	

Abbreviations: AML, acute myeloid leukemia; Bu, Busulfan; Cy, Cyclophosphamide; Cyto, cytogenetics; IQR, interquartile range; KPS, Karnofsky performance score (KPS); Mel, melphalan; TBI, total body irradiation; Tx, transplantation.

### 3.2 Transplantation outcomes

**FIGURE 1:**
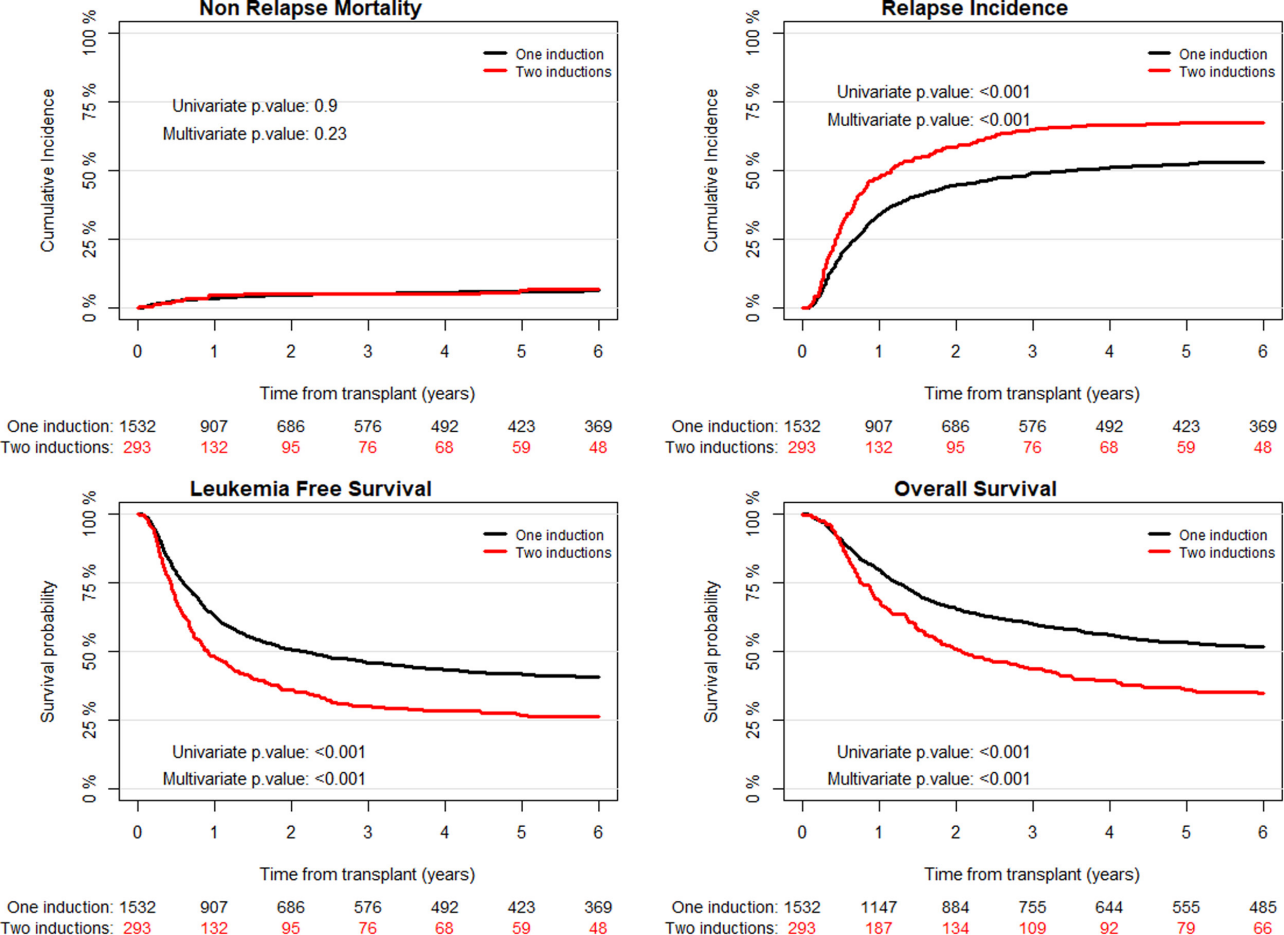
Autologous transplantation outcome – Non‐relapse mortality (NRM), relapse incidence (RI), leukemia‐free survival (LFS) and overall survival (OS) in patients with AML with one and two induction courses.

The incidence of day 30 neutrophil recovery was 96% and 96.5% in patients receiving 1 versus 2 induction courses, respectively (Table 2). Two‐ and 5‐year NRM did not differ significantly between patients achieving CR1 with 2 versus 1 chemotherapy courses: 5.3% versus 4.8% and 6.2% versus 6.0%, respectively (*p* = 0.9) (Table 2). Two‐ and 5‐year RI was higher: 58.6% versus 44.7% and 67.2% versus 52.3% (*p* < 0.001), while 2‐ and 5‐year LFS and OS were lower for patients achieving CR1 with 2 versus 1 course of chemotherapy: 36.1% versus 50.6% and 41.7% versus 26.6% (*p* < 0.001) and 51% versus 65.5% and 36.2% versus 53.3% (*p* < 0.001), respectively (Table 3, Figure 1).

**TABLE 2:** Transplantation outcome

	All the patients	1 induction	2 inductions
Clinical parameter	Estimation (95%CI)	Estimation (95%CI)	Estimation (95%CI)
Median FU (y)	7.7 (7–8.6)	7.9 (7.4–8.4)	7.7 (7–8.6)
Neutrophil recovery (30 d)	96.5 (93.5–98.1)	96 (94.9–96.9)	96.5 (93.5–98.1)
NRM (2 y)	5.3 (3.1–8.4)	4.8 (3.7–5.9)	5.3 (3.1–8.4)
NRM (5 y)	6.2 (3.7–9.5)	6 (4.8–7.3)	6.2 (3.7–9.5)
RI (2 y)	58.6 (52.5–64.2)	44.7 (42.1–47.2)	58.6 (52.5–64.2)
RI (5 y)	67.2 (61.2–72.5)	52.3 (49.6–54.9)	67.2 (61.2–72.5)
LFS (2 y)	36.1 (30.4–41.7)	50.6 (48–53.1)	36.1 (30.4–41.7)
LFS (5 y)	26.6 (21.4–32)	41.7 (39.1–44.3)	26.6 (21.4–32)
OS (2 y)	51 (44.9–56.7)	65.5 (63–67.9)	51 (44.9–56.7)
OS (5 y)	36.2 (30.4–42)	53.3 (50.6–56)	36.2 (30.4–42)

Abbreviations: d, day; FU, follow up; LFS, leukemia, free survival; NRM, non, relapse mortality; OS, overall survival; PMN, polymorphonuclear leukocytes; RI, relapse incidence; y, year.

**TABLE 3:** Univariate analysis

(A)‐2 y					
Variable	Modalities	2 y OS	2 y LFS	2 y RI	2 y NRM
Total number of induction to reach CR1	One	65.5 [63–67.9]	50.6 [48–53.1]	44.7 [42.1–47.2]	4.8 [3.7–5.9]
	Two	51 [44.9–56.7]	36.1 [30.4–41.7]	58.6 [52.5–64.2]	5.3 [3.1–8.4]
	*p* value	<0.001	<0.001	<0.001	0.9
Patient sex	Male	62.9 [59.7–66]	46.4 [43.2–49.6]	49.3 [46–52.4]	4.3 [3.1–5.7]
	Female	63.5 [60–66.7]	50.4 [46.9–53.9]	44.1 [40.6–47.5]	5.5 [4–7.2]
	*p* value	0.49	0.02	0.03	0.89
KPS	<90	59.7 [53.7–65.1]	46.6 [40.7–52.2]	48.4 [42.5–54]	5.1 [3–8]
	> = 90	66 [62.3–69.4]	50.4 [46.6–54.1]	46 [42.2–49.7]	3.7 [2.4–5.3]
	*p* value	0.13	0.54	0.7	0.92
Cyto AML classification	good	80.3 [74.8–84.6]	63.1 [57–68.7]	34.3 [28.6–40]	2.6 [1.2–5]
	intermediate	61.1 [58.2–63.8]	46.9 [44–49.8]	48.3 [45.4–51.2]	4.7 [3.6–6]
	poor	46.5 [37–55.4]	32.7 [24.3–41.4]	61.2 [51.5–69.5]	6.1 [2.7–11.5]
	NA/failed	63.1 [55.6–69.6]	45.5 [38.2–52.5]	46.5 [39.2–53.6]	8 [4.7–12.4]
	*p* value	<0.001	<0.001	<0.001	0.03

(B)‐5 y					
Variable	Modalities	5 y OS	5 y LFS	5 y RI	5 y NRM
Total nbr of induction to reach CR1	One	53.3 [50.6–56]	41.7 [39.1–44.3]	52.3 [49.6–54.9]	6 [4.8–7.3]
	Two	36.2 [30.4–42]	26.6 [21.4–32]	67.2 [61.2–72.5]	6.2 [3.7–9.5]
	*p* value	<0.001	<0.001	<0.001	0.9
Patient sex	Male	50.4 [47.1–53.7]	37.5 [34.3–40.7]	56.7 [53.4–59.9]	5.7 [4.3–7.4]
	Female	50.6 [46.9–54.2]	41.3 [37.8–44.8]	52.3 [48.7–55.8]	6.4 [4.8–8.2]
	*p* value	0.49	0.02	0.03	0.89
KPS	<90	46.3 [40.2–52.2]	38.9 [33.2–44.7]	55 [49–60.7]	6 [3.6–9.2]
	>= 90	54.7 [50.7–58.6]	40.4 [36.6–44.2]	54.9 [50.9–58.6]	4.7 [3.3–6.5]
	*p* value	0.13	0.54	0.7	0.92
Cyto AML classification	Favorable	72.6 [66.4–77.8]	59.4 [53.1–65.2]	38 [32–43.9]	2.6 [1.2–5]
	interm	47.8 [44.8–50.7]	36.5 [33.6–39.3]	57.6 [54.6–60.4]	6 [4.7–7.4]
	Adverse	32.5 [23.8–41.5]	26 [18.2–34.4]	67.9 [58.3–75.8]	6.1 [2.7–11.5]
	NA/failed	48.9 [41.2–56.1]	38.2 [31–45.3]	50.8 [43.3–57.8]	11 [7–16.1]
	*p* value	<0.0001	<0.0001	<0.0001	0.03

Abbreviations: AML, Acute myeloid leukemia; CR1, first complete remission; Cyto, cytogenetics; KPS, Karnofsky performance status; LFS, leukemia‐free survival; NA, not available; NRM, non, relapse mortality; OS, overall survival; RI, relapse incidence; y, year.

### 3.3 Multivariate analysis

Table 4 shows the results of the multivariate analysis (MVA). Relapse incidence was significantly higher in patients achieving remission after 2 induction courses in comparison to the patients who needed only 1 induction course (HR = 1.46;95% CI: 1.25–1.72; *p* < 0.001) leading to significantly lower LFS (HR = 1.42; 95% CI: 1.22–1.66; *p* < 0.001) and OS (HR = 1.48; 95% CI: 1.25–1.75, *p* < 0.001), respectively. NRM was similar between the two cohorts (2 vs. 1 induction courses) (HR = 1.31; 95% CI: 0.81–2.10, *p* = 0.27). Adverse‐ compared to favorable‐risk cytogenetics and older age were additional significant prognostic factors for post‐transplant outcomes including RI, NRM, LFS, and OS. Cytogenetics (intermediate‐ vs. favorable‐risk) was an additional prognostic factor for RI, LFS, and OS. Finally, female gender was a prognostic factor for RI and LFS; and more recent year of the transplant was a prognostic factor for RI and OS (Table 4).

**TABLE 4:** Multivariate analysis

Variables	Modalities	OS		LFS		RI		NRM	
		HR (95% CI)	*p* value	HR (95% CI)	*p* value	HR (95% CI)	*p* value	HR (95% CI)	*p* value
Total number of induction	One	1		1		1		1	
	Two	1.48 (1.25–1.75)	<0.001	1.42 (1.22–1.66)	<0.001	1.46 (1.25–1.72)	<0.001	1.31 (0.81–2.1)	0.27
Cyto AML classification	Good	1		1		1		1	
	Intermediate	1.99 (1.56–2.55)	<0.001	1.7 (1.38–2.09)	<0.001	1.72 (1.39–2.13)	<0.001	1.75 (0.9–3.39)	0.1
	Poor	3.07 (2.23–4.24)	<0.001	2.47 (1.85–3.29)	<0.001	2.53 (1.88–3.41)	<0.001	2.53 (1.05–6.14)	0.04
	NA/failed	1.87 (1.38–2.54)	<0.001	1.63 (1.25–2.12)	<0.001	1.55 (1.17–2.05)	0.002	2.74 (1.29–5.8)	0.008
Patient sex	Male	1		1		1		1	
	Female	0.92 (0.81–1.05)	0.21	0.85 (0.75–0.96)	0.008	0.85 (0.74–0.96)	0.01	0.91 (0.64–1.29)	0.6
Year at transplant (by 5 y)		0.86 (0.79–0.93)	<0.001	0.94 (0.87–1.01)	0.07	0.93 (0.86–0.99)	0.03	0.99 (0.82–1.21)	0.94
Age at transplant (by 10 y)		1.22 (1.15–1.29)	<0.001	1.13 (1.07–1.18)	<0.001	1.08 (1.02–1.13)	0.004	1.59 (1.36–1.87)	<0.001

Abbreviations: Cyto, cytogenetics; HR, hazard ratio; LFS, leukemia‐free survival; NA, not available; NRM, non‐relapse mortality; OS, overall survival; RI, relapse incidence; y, year.

### 3.4 Causes of death

A total of 733 (48%) and 185 (63%) patients in the 1‐ and 2‐induction cohorts, respectively, died during the study period (Table 5). The original disease was the main cause of death, being 88% and 90% of the deaths of patients receiving 1 versus 2 induction courses, respectively. Other causes of death were infections, secondary malignancies, and other transplant‐related causes (Table 5).

**TABLE 5:** Causes of death

Cause of death	One induction	Two inductions
Original disease	628	167
Infection	35	5
Secondary malignancy	20	1
GVHD of consecutive alloHSCT	7	1
Cardiac toxicity	4	1
Gastro intestinal toxicity	1	0
Hemorrhage	3	4
Hepatic toxicity	1	1
Lymphoproliferative disorder	0	1
MOF	2	1
Pulmonary toxicity	3	0
Renal failure	2	1
Rejection	1	1
VOD	2	0
HSCT related (not specified)	1	0
Other	6	1
Missing	17	0

Abbreviations: MOF, multi organ failure; VOD, veno‐ occlusive disease of the liver.

## 4 DISCUSSION

Post remission consolidation with ACT in adult patients with AML has several advantages over other therapeutic options including both the very low early as well as long‐term transplant‐related mortality and transplant‐related complications^8^ and the absence of graft‐versus‐host disease (GVHD) which is still the main obstacle to successful allogeneic transplantation.^1^ In addition, ACT may be followed by targeted therapies and post‐transplant maintenance therapy and may serve as a safe and attractive platform for cellular immunotherapies as the ACT patients are free of immunosuppressive drugs.^25^, ^26^ One of the key issues in the ACT field is to define prognostic and predictive factors that will help in defining and selecting the AML patients that will benefit most from ACT sparing them the risk of allogeneic transplantation. Such prognostic factors historically include the AML‐related factors such as FAB‐ acute promyelocytic leukemia and favorable‐risk cytogenetics, response to front‐line therapies, but mainly the achievement of MRD negativity and factors related to the graft such as cell dose, and to the recipient.^3^, ^4^, ^5^, ^27^, ^28^ Shouval et al. recently added to this list of prognostic factors two frequent AML‐related mutations: FLT3–ITD and NPM1 demonstrating that molecular subtype is a strong predictor of LFS, OS, and relapse and that AML patients with intermediate‐risk cytogenetics expressing the FLT3‐ITD^neg^/NPM1^mut^ mutation phenotype experience favorable outcomes when autografted in CR1 with a 5‐year LFS of 62% and OS of 74% indicating that ACT is an attractive option for these patients.^7^ Shouval et al. also recently developed a prognostic model for estimating the probability of LFS and OS after ACT in patients with AML in CR1 undergoing transplantation as post‐remission therapy based on age, cytogenetics, and FLT3‐ITD status integrating them into a nomogram (the Auto‐AML score) to be used for the estimation of outcomes after ACT demonstrating that patients with low scores do exceedingly well after ACT.^6^ In the current study, we added the number of induction courses needed to achieve CR1 as a possible additional prognostic factor for long‐term ACT outcome in AML patients demonstrating that achieving a CR1 after the first induction course is an important predictor of transplantation outcome. Relapse incidence was statistically significantly higher in the leukemic patients who needed 2 lines of chemotherapy in order to clear their leukemic blasts and reach CR in comparison to their counterparts needing 1 line of chemotherapy, respectively. The lower relapse rate was translated into a significant LFS and OS advantage for AML patients needing only 1 course. The fact that NRM was similar between the leukemic patients who got 1 or 2 lines of induction chemotherapy indicates that the better outcome seen in the former group is not due to a higher cumulative dose of chemotherapy leading to increased transplant‐related toxicity and death in those patients who received 2 induction courses. It is conceivable that achieving a CR after a 2 versus 1 course of chemotherapy is indicative of more aggressive biology of the leukemia and may be a surrogate marker for leukemic resistance. Our data emphasize the recent emerging concept in hematology malignancies in general and AML in particular that it is a quality of CR rather than its one‐point static achievement that is important in the malignancy treatment paradigm.^29^, ^30^ It is possible that the quality of the remission was superior in patients that received only 1 line of induction therapy which translated into a better outcome. In agreement with this premise, it was recently shown that AML patients attaining CR with incomplete count recovery (CRi) prior to HSCT had an inferior 5‐year probability of survival in comparison to patients attaining CR with complete count recovery i.e., 24.4% versus 51.3%, respectively (*p* < 0.001)^31^; HR 2.01; 95% CI: 1.24–3.25; *p* = 0.005.^32^ The improved transplantation outcome in AML patients achieving CR1 after 1 versus 2 inductions was previously shown by others as well as our group for HSCT from a sibling and unrelated as well as haploidentical donors.^16^, ^17^, ^18^, ^19^ However, this phenomenon is probably more important in the autologous versus the allogeneic setting as there is no GVL effect in the former setting and thus the anti‐leukemic effect and the curative potential of the ACT are based only on the high dose chemotherapy and therefore the depth and quality of the remission are crucial and probably even more important than in the allogeneic setting. In support of this likely explanation is on the one hand our historical finding that marrow purging with mafosfamide prior to ACT was shown to benefit essentially slow remitters and not rapid remitters^15^, ^33^ and on the other hand, our more recent finding that in cord blood transplants were the GVL effect may be stronger as compared to allogeneic transplants from matched and mismatched unrelated donors,^34^ we did not observe any difference in transplantation outcome between AML patients attaining CR1 with 1 versus 2 chemotherapy lines.^35^ Our findings that ACT outcome is unsatisfactory with very high (close to 70%) 5‐year RI and low 5‐year LFS and OS (approximately 30%) in a defined subgroup of AML patients (those needing 2 induction courses to achieve CR1) in spite of being in CR1 and not in a more advanced disease status at ACT, has important clinical implications. This subgroup of AML patients should probably be offered an allogeneic SCT rather than an ACT. The number of induction courses to achieve CR is an additional prognostic factor to be incorporated into the decision making with respect to tailored post remission therapy for AML patients.

Adverse cytogenetics, increasing age, gender, and year of transplant were additional prognostic factors for ACT outcome, in accordance with previous studies.^1^, ^9^, ^10^, ^11^ Our study suffers from several limitations due to the fact that it is a transplant registry‐based study. These limitations include the possibility of unavailable data that could not be considered, mainly missing molecular and MRD information which dictate our definition of CR as <5% blasts in bone marrow, while the updated definition of CR includes MRD and additional parameters.^20^, ^21^ Moreover, we cannot exclude confounding due to the lack of pre‐transplantation data. Finally, the AML treatment landscape is evolving with the recently approved novel drugs, including azacytidine, decitabine, venetoclax, and Vyxeos (CPX‐351) and our findings are applicable only to AML patients receiving the conventional 7 + 3 induction regimen.^36^ Also, as our study is not an onset‐to‐treat analysis, patients who could not be mobilized are naturally not included in the analysis. On the other hand, our study involves a relatively large AML patient cohort and reflects the real‐life current clinical scenario in multiple European transplant centers. In conclusion, in our study, we demonstrated that patients with AML undergoing ACT in the first remission achieved after 2 induction courses, had a very high 5‐year relapse rate resulting in a significantly inferior LFS and OS in comparison to patients achieving CR after 1 course of chemotherapy. These patients should most probably in accordance with the results of the GIMEMA 1013 risk‐adapted MRD‐directed therapy trial^37^ either receive additional consolidation courses and be monitored to reach a status of undetectable MRD prior to ACT or be offered allogeneic transplantation to reduce high relapse rates and improve outcome. The number of inductions to achieve CR should be an additional factor in the AML patient prognostication algorithm and the physician's decision for the preferred post‐remission therapy tailored to the individual patient.

### AUTHOR'S CONTRIBUTION

A.N wrote the manuscript, designed the study, and interpreted the data. J.E, M.L designed the study, performed the statistical analyses, interpreted the data, and edited the manuscript, D.B; W.A; SM. T; D.W; A.P; GV. G; MT. R; TG. D; A.H; F.L; NC. G reviewed the manuscript and provided clinical data. All authors approved the final version of the manuscript.

## CONFLICT OF INTEREST

The authors declare no competing financial interests.

## ETHICS APPROVAL AND CONSENT TO PARTICIPATE

The scientific boards of the ALWP of the EBMT approved this study.

## CONSENT FOR PUBLICATION

Not applicable.

## Supporting information


Appendix S1

## Data Availability

AN, JE, CG, ML and MM had full access to all study data (available upon data specific request).
