# Supplementary material for: Autologous stem cell transplantation (ASCT) for acute myeloid leukemia in patients in first complete remission after one versus two induction courses: A study from the ALWP of the EBMT
Source: Cancer Med. 2022 Jul 26;12(2):1482–91. doi: 10.1002/cam4.5039 (PMC9883552; doi:10.1002/cam4.5039)
Supplement: Supplementary file 1 — Appendix S1 [file CAM4-12-1482-s001.docx]

**Supplementary Appendix: Contributing Centers**

Programme de Transplantation & Therapie Cellulaire, Centre de Recherche en Cancérologie de Marseille, Institut Paoli Calmettes, Marseille, France ; ¨Tor Vergata¨ University of Rome, Stem Cell Transplant Unit, Policlinico Universitario Tor Vergata, Rome, Italy; Univ. La Sapienza, Dip. Biotecnologie Cellulari ed Ematologia, Rome, Italy; First Affiliated Hospital of Soochow University, Department of Hematology, Suzhou, China; CHU Bordeaux, Hôpital Haut-leveque, Pessac, France; University Hospital Maastricht, Dept. Internal Med.Hematology /Oncology, Maastricht, The Netherlands; CHRU BRABOIS, Vandoeuvre les Nancy, France; Hopital Saint Antoine, Department of Hematology, Paris, France; Oslo University Hospital, Rikshospitalet, Clinic for Cancer Medicine, Hematology Dept., Section for Stem Cell Transplantation, Oslo, Norway; CHU - Institut Universitaire du Cancer Toulouse, Oncopole, Toulouse, France; University Hospital Gasthuisberg, Dept. of Hematology, Leuven, Belgium; Peking University People´s Hospital, Institute of Haematology, Xicheng District, Beijing, China; Nijmegen Medical Centre, Department of Hematology, Nijmegen, The Netherlands; CHU Nantes, Dept. D`Hematologie, Nantes, France; Ospedale La Maddalena - Dpt. Oncologico, Unità Operativa di Oncoematologia e, Trapianto di Midollo, Palermo, Italy; Cardarelli Hospital, Division of Hematology && SCT Unit, Napoli, Italy; Gazi University Faculty of Medicine, Hematology, Ankara, Turkey; Hospital U. Marqués de Valdecilla, Servicio de Hematología-Hemoterapia, Santander, Spain; S.S.C.V.D Trapianto di Cellule Staminali, A.O.U Citta della Salute e della Scienza di Torino, Torino, Italy; ICO-Hospital Universitari Germans Trias i Pujol, Cattedra e Servizio di Ematologia, Badalona, Spain; Hospital Universitario Central de Asturias, Avenida de Roma S/N, Oviedo, Spain; Techniciens d`Etude Clinique suivi de patients greffes, Nouvel Hopital Civil, Strasbourg, France; Institut Jules Bordet, Experimental Hematology, Brussels, Belgium; Hopital d`Enfants, CHU de Dijon, Service Hematologie Adultes, Dijon, France; Hospital Clínico, Servicio de Hematología, Salamanca, Spain; Medisch Spectrum Twente, Postbus 50000, Enschede, The Netherlands; Klinikum Nuernberg, 5. Medizinische Klinik, BMT-Unit, Nuernberg, Germany ; CHRU, Service des Maladies du Sang, Angers, France ; Hopital Jean Minjoz, Service d`Hématologie, Besancon, France; Hospital son LLatzer, Cr. Manacor, Km 4, Palma Mallorca, Spain; Ospedale Santa Maria Goretti, Ematologia, Latina, Italy; Haga Hospital (Leyenburg), Dept. Hematology, The Hague, The Netherlands; Hosp. Reina Sofia, Córdoba Hospital, Department of Hematology, Cordoba, Spain; Centre Hospitalier Lyon Sud, Pavillon Marcel Bérard -Bat 1G, Service Hematologie, Lyon, France; ICO – Hospital Duran i Reynals, Av. Gran Vía 199-203, L`Hospitalet de Llobregat, Barcelona, Spain; Unita Operativa di Ematologia e Trapianto di cellule staminali, Presidio Ospedaliero Vito Fazzi, Lecce, Italy; Hospital Vall d`Hebron, Unidad de Adultos, Barcelona, Spain; Medical University of Gdansk, University Hospital, Dept. of Haematology and Transplantology, Gdansk, Poland; Cliniques Universitaires St. Luc, Dept. of Haematology, Brussels, Belgium; CHU Grenoble Alpes - Université Grenoble Alpes, Service d`Hématologie, CS 10217, Grenoble, France; CHRU Limoges, Service d`Hématologie Clinique, Limoges, France; University Hospital Bern, Paediatric Hematology/Oncology, Bern, Switzerland; Hopital La Miletrie, Head of the Bone Marrow TransplantUnit, Clinical Hematology, Poitiers, France; CHU ESTAING, Service d’hématologie clinique Adulte et pédiatrie, Clermont Ferr, France; Azienda Ospedaliera Universitaria Careggi, Cell Therapy and Transfusion Medicine Unit, Firenze, Italy; CHU Nice - Hôpital de l`ARCHET I, Hematologie Clinique, Nice, France; Institute of Hematology and Transfusion Medicine, Warsaw, Poland; Hôpital Robert Debre, Hematology Department, Reims, France; Erasmus MC Cancer Institute, University Medical Center Rotterdam, Department of Hematology, Rotterdam, The Netherlands; Dél-pesti Centrumkórház –, Országos Hematológiai és Infektológiai Intézet, Dept. Haematology and Stem Cell Transplant, Budapest, Hungary; University Hospital, Hematology, Basel, Switzerland; Leiden University Hospital, BMT Centre Leiden, Leiden, The Netherlands; Unità Operativa di Ematologia, Ospedale Civile, Ravenna, Italy; U.O. Ematologia con Trapianto, Azienda Ospedaliero Universitaria Policlinico Bari, Bari, Italy; University of Liege, Dept. of Hematology, CHU Sart-Tilman, Liege, Belgium; H SS. Antonio e Biagio, Haematology Department, Alessandria, Italy; Klinik fuer Innere Medzin III, Universitätsklinikum Ulm, Ulm, Germany; Ospedale San Gerardo, Clinica Ematologica dell`Universita Milano-Biocca, Monza, Italy; University Hospital, Department of Hematology and Transfusiology, Bratislava, Slovakia; Vilnius University Hospital Santaros Klinikos, Haematology, Oncology & Transfusion Center, Vilnius, Lithuania; Centre Hospitalier Universitaire de Rennes, Service d`Hematologie Clinique Adulte, Rennes, France; Fondazione IRCCS - Ca’ Granda, Ospedale Maggiore Policlinico IRCCS, Milano, Italy; L´Hôpital Erasme, Dept. of Haematology, Brussels, Belgium; Universitair Ziekenhuis Brussel, Division of Clinical Hematology, Brussels, Belgium; ASST Papa Giovanni XXIII, Hematology and Bone Marrow Transplant Unit, Bergamo, Italy; Charles University Hospital, Dept. of Hematology/Oncology, Pilsen, Czech Republic; Hôpital E Muller, Service d´Hématologie Clinique, Unité de Greffes - Unité de Thérapie Cellulaire, 2, Mulhouse, France; Hôpital Necker, Service Hematologie Adulte, Paris, France; Hopital Bretonneau, Service d`Oncologie Médicale, Tours, France; Skanes University Hospital, Dept. of Hematology, Lund, Sweden; ZNA, Lange Beeldekensstraat 267, Antwerp, Belgium; Rambam Medical Center, Dept. of Hematology & BMT, Haifa, Israel; Klinikum Bremen-Mitte, Hämatologie / Onkologie, Klinik für Innere Medizin, Bremen, Germany; Samodzielny Publiczny, Szpital Kliniczny Nr 1 w Lublinie, Klinika  Hematoonkologii i Transplantacji Szpiku, Lublin, Poland; Fundació Institut d`Investigació Sanitària Illes Balears – IdISBa, Hospital Universitari Son Espases. Edifici “S”. 1ª Planta, Palma Mallorca, Spain; University of Saarland, University Hospital, Dept. of Internal Med., BMT Unit, Homburg, Germany; Universitaetsklinikum Dresden, Medizinische Klinik und Poliklinik I, Dresden, Germany; Ospedale San Raffaele s.r.l., Haematology and BMT, Milano, Italy; Karolinska University Hospital, Dept. of Hematology, Stockholm, Sweden; Institut de Cancerologie Lucien Neuwirth, Service d`Hematologie Clinique, Saint Etienne, France; Hopital Cochin, Paris, France; Hannover Medical School, Department of Haematology, Hemostasis, Oncology, and Stem Cell Transplantation, Hannover, Germany; C.H.R.U de Brest, Service Onco-Hematologie, Brest, France; University Regensburg, Dept. of Hematology and Oncology, Regensburg, Germany; Ospedale Policlinico, Programma di Trapianto Emopoietico Misto e Metropolitano Di Catania, Catania, Italy; ZSIS Universitaetsklinikum Knappschaftskrankenhaus Bochum GmbH, Medizinische Klinik - Hämatologie und Onkologie, Bochum, Germany; Fondazione IRCCS Policlinico San Matteo, Pavia, Italy; Kantonsspital Aarau, Center of Onc/Hematology & Transfusion Medicine, Aarau, Switzerland; VU University Medical Center, Department of Hematology (Br 250), Amsterdam, The Netherlands; Hospital C. Panico, Hematology, Tricase Lecce, Italy; Constantiaberg Medi-Clinic, Cape Haematology and Bone Marrow Transplant Unit, Cape Town, South Africa; Azienda Ospedali Riuniti di Ancona, Department of Hematology, Ancona University, Ancona, Italy; Hospital Gregorio Marañón, Sección de Trasplante de Medula Osea, Madrid, Spain; Hospital Guglielmo da Saliceto, Oncology and Hematology Department, Piacenza, Italy; Onco-Ematologia Pediatrica, Centro Trapianti Cellule Staminali, Ospedale Infantile Regina Margherita, Torino, Italy; EVK Hamm, Onkologische Dokumentation, Hamm, Germany; HUCH Comprehensive Cancer Center, Stem Cell Transplantation Unit, Helsinki, Finland; NADACE HAIMOM, University Hospital, Department of Haemato-Oncology, Olomouc, Czech Republic; Hospital Universitario Donostia, Paseo Dr Beguiristain 107-116, San Sebastian, Spain; Antwerp University Hospital (UZA), Dept. of Hematology, Antwerp Edegem, Belgium; Klinikum Chemnitz gGmbH, Innere Medizin III, Chemnitz, Germany; Evangelische Diakonie, Haematologie/Internisti, Evangelische Diakonissenanstal.sche Onk., Bremen, Germany; Sana Klinikum Hameln-Pyrmont, Abt. Haem/Onkologie, Hameln, Germany; CHU de Lille, LIRIC, INSERM U995, Université de Lille, Lille, France; Hospital Clínico de Valencia, Servicio de Hematología, Valencia, Spain; Spedali Civili - Brescia, Hematology Division, Department of Medical Oncology, Brescia, Italy; King Faisal Specialist Hospital & Research Centre, Oncology (Section of Adult Haematolgy/BMT), Riyadh, Saudi Arabia; Azienda Ospedaliero Universitaria di Modena Policlinico, Ematologia, Modena, Italy; Grande Ospedale Metropolitano Bianchi Melacrino Morelli - Centro Unico Trapianti A. Neri, Via G. Melacrino, 21, Reggio Calabria, Italy; Az. Ospedaliera S. Croce e Carle, Division of Hematology, Cuneo, Italy; University Med. Center, Department of Hematology, Ljubljana, Slovenia; Staedtisches Klinikum Braunschweig, Medizinische Klinik III, Innere Med. - Hämatologie und Onkologie, Braunschweig, Germany; Hanuschkrankenhaus, 3rd Dept. of Internal Med. / Hematology, Vienna, Austria; University Hospital, Collegium Medicum UMK, Pediatric Hematology and Oncology, Bydgoszcz, Poland; Centro Trapianti Unico Di CSE Adulti e Pediatrico A. O Brotzu, Via Edward Jenner, 1, P.O. R. Binaghi, Cagliari, Italy; Istituto Di Ematologia, Universita` Di Sassari, Sassari, Italy; Klinikum Ludwigshafen GmbH, Haem./Internist. Onk., Ludwigshafen, Germany; Imperial College, Department of Haematology, Hammersmith Hospital, London, United Kingdom; University Medical Centre, Dept. of Haematology, Utrecht, The Netherlands; Univ. of Parma, Cattedra di Ematologia, Centro Trapianti Midollo Osseo, Parma, Italy; U.O.S.A Centro Trapianti e Terapia Cellulare, Azienda Ospedaliera Universitaria Senese, Policlinico S.Maria alle Scotte, Siena, Italy; U.O.D Trapianti di midollo osseo, A.O.R Villa Sofia-Cervello, Via Trabucco 180, Palermo, Italy; Hospital Clínico, Dept. of Hematology, Zaragoza, Spain; Hospital Clinico Universitario, Servicio de Hematología, S de Compostela, Spain; Jessa Ziekenhuis, Dept. of Hematology, Hasselt, Belgium; Shariati Hospital, Hematology-Oncology and BMT Research, Teheran, Iran; Silesian Medical Academy, Univ. Dept. of Haematology and BMT, Katowice, Poland; First State Pavlov Medical University of St. Petersburg, Raisa Gorbacheva Memorial Research Institute for Paediatric Oncology, Hematology, and Transplantation, St Petersburg, Russia; Centro Medico Teknon, Consultorios Vilana, Barcelona, Spain; Hospital Universitario Sanitas La Zarzuela, Hematology, Madrid, Spain; University of Freiburg, Dept. of Medicine -Hematology, Oncology, Freiburg, Germany; Hospital dos Capuchos, Servico de Hematologia, Lisboa, Portugal; National Hospital of Haematological Diseases, Bone Marrow Transplant, Sofia, Bulgaria; C.H. de la Côte Basque, Service d`Hématologie, Bayonne, France; Medicana International Hospital Istanbul, Bone Marrow Transplant Unit, Istanbul, Turkey; CHU Lapeyronie, Département d`Hématologie Clinique, Montpellier, France; Centre Antoine Lacassagne, Service d`Hemato-Oncologie, Nice, France; Evangelisches Klinikum Bethel, Klinik für Innere Medizin, Hämatologie-, Onkologie und Palliativmedizin, Bielefeld, Germany; HELIOS Klinikum Wuppertal GmbH, Med. Klinik I, Hamatologie/Onkologie, Wuppertal, Germany; Universitaet Bonn, Medizinische Klinik III, Bonn, Germany; Klinikum Bayreuth GmbH, Medizinische Klinik IV, Hämatologie und Internistische Onkologie, Bayreuth, Germany; Universitaetsmedizin Mannheim, III. Medizinische Klinik, Einheit für Stammzelltransplantation, Mannheim, Germany; Robert_Bosch_Krankenhaus, Abt. Hämatologie / Onkologie, Stuttgart, Germany; Asklepios Klinik St. Georg, Department of Haematology, Hamburg, Germany; Centro di Riferimento Oncologico, C.R.O. IRCCS Aviano, Aviano, Italy; Ospedale Umberto I, U.O. Medicina Interna Oncoematologia, Salerno, Italy; St. Antonius Hospital, Dept. of Internal Medicine, Nieuwegein, The Netherlands; Turku University Hospital, TD7 (Stem Cell Transplant Unit), Turku, Finland; Hospital Santa Creu i Sant Pau, Hematology Department, Barcelona, Spain; The Trustee of London Clinic, Stem Cell Transplant Unit, London, United Kingdom; Belfast City Hospital, Dept. of Haematology, Belfast, United Kingdom; Research Committee - University of Patras, University Campus - Building A, Patras, Greece; Ospedale S. Camillo-Forlanini, Dept. of Hematology and BMT, Rome, Italy; ASST GRANDE OSPEDALE METROPOLITANO NIGUARDA, Hematology Department, Milano, Italy; Goethe-Universitaet, Medizinische Klinik II, Hämatologie, Medizinische Onkologie, Frankfurt Main, Germany; Hospital San Maurizio, Dept. of Hematology - BMT Unit, Bolzano, Italy; Kaplan Medical Centre, Hematology Institute, Rehovot, Israel; GHDC, Department of Hemato-Oncology, Charleroi, Belgium; Westpfalz-Klinikum GmbH, Medizinische Klinik I, Kaiserslautern, Germany; Complejo Hospitalario de A Coruña, Hematologia (Planta 11ª), La Coruna, Spain; Gaziantep University Medical School, Division of Haematology, Gaziantep, Turkey; Hospital de León, Haematology Department, Leon, Spain; ALBERTS CELLULAR THERAPY, Netcare Pretoria East Hospital, Pretoria, South Africa; A.Z. Sint-Jan, Dept. of Hematology, Brugge, Belgium; HELIOS Klinikum Duisburg, An der Abtei 7-11, Duisburg, Germany; Hospital de Gran Canaria `Dr Negrin`, Servicio de Hematología y Hemoterapia, Las Palmas, Spain; Civic Hospital, Dept. of Oncology, Noale, Italy; Dr. Horst-Schmidt-Kliniken, Dept of Medicine III, Hematology / Oncology, Wiesbaden, Germany; Baskent University Hospital, Haematology Division, BMT Unit, Haemaology Reserach Laboratory, Training & Medical, Adana, Turkey; Klinik fuer Knochenmarktransplantation, und Hämatologie/Onkologie GmbH, Idar Oberstein, Germany; University Hospital Eppendorf, Bone Marrow Transplantation Centre, Hamburg, Germany; University of Milano, Istituto Nazionale dei Tumori, Hematology - Bone Marrow Transplantation Unit, Milano, Italy; Ege University Medical School, Dept. of Hematology, Bornova, Izmir, Turkey; University of Debrecen Clinical Center, Department of Internal Medicine, Debrecen, Hungary; Klinikum Harlaching, Department of Haematology & Oncology, Munich, Germany; Hospital Universitario Puerta del Mar, Servicio de Hematología, Cadiz, Spain; Ysbyty Gwynedd, Dept of Clinical Haematology & Oncology, Alaw Unit, Bangor, United Kingdom; Ghent University Hospital, Haematology, Gent, Belgium; University of Napoli, `Federico II` Medical School, Division of Hematology, Napoli, Italy; Fondazione S. Maugeri, Div. Oncologia Medica, Pavia, Italy; Military Institute of Health Services BMT Unit, Bone Marrow Transplantation Unit, Warsaw, Poland; Centre Hospitalier Universitaire Vaudois, Centre Hospitalier Universitaire Vaudois, División of Hematology, Lausanne, Switzerland; Ospedale Regionale Bellinzona e Valli, Istituto Oncologico della Svizzera italiana, Unità Trapianti, Ist. Oncologico della Svizzera It, Bellinzona, Switzerland; Ospedale San Carlo, Dip. Ematologia, Potenza, Italy; Azienda Ospedaliero-Universitaria Maggiore della Carità, SCDU Ematologia, Amedeo Avogadro University of Eastern Piedmont, Novara, Italy; ASST Valle Olona - Ospedale di Circolo di Busto Arsizio, Ematologia, Busto Arsizio, Italy; Hopital Pasteur, Service d`onco hematologie, Colmar, France; Hôpital St Vincent de Paul, Hématologie, Lille, France;
